# The Effect of Phytogenic Additive in Broiler’s Diet on Production Results, Physicochemical Parameters, and the Composition of Volatile Organic Compounds of Broiler Meat Assessed by an Electronic Nose System

**DOI:** 10.3390/ani14162428

**Published:** 2024-08-22

**Authors:** Monika Michalczuk, Paulina Abramowicz-Pindor, Jakub Urban, Damian Bień, Patrycja Ciborowska, Arkadiusz Matuszewski, Anna Zalewska, Eliza Opacka, Iwona Wojtasik-Kalinowska

**Affiliations:** 1 Department of Animal Breeding, Institute of Animal Sciences, Warsaw University of Life Sciences, Ciszewskiego 8, 02–786 Warsaw, Poland; monika_michalczuk@sggw.edu.pl (M.M.); jakub_urban@sggw.edu.pl (J.U.); patrycja_ciborowska@sggw.edu.pl (P.C.); anna_zalewska@sggw.edu.pl (A.Z.); eliza_opacka@sggw.edu.pl (E.O.); 2 Department of Research and Development, AdiFeed Sp. z o.o., Chrzanowska 15, 05-825 Grodzisk Mazowiecki, Poland; paulina.abramowicz-pindor@adifeed.pl; 3 Division of Animal Nutrition, Institute of Animal Sciences, Warsaw University of Life Sciences, Ciszewskiego 8, 02–786 Warsaw, Poland; damian_bien1@sggw.edu.pl; 4 Department of Animal Environment Biology, Institute of Animal Sciences, Warsaw University of Life Sciences, Ciszewskiego 8, 02-786 Warsaw, Poland; 5 Department of Technique and Food Development, Institute of Human Nutrition Sciences, Warsaw University of Life Sciences, Nowoursynowska 159 C, 02-776 Warsaw, Poland; iwona_wojtasik-kalinowska@sggw.edu.pl

**Keywords:** broiler chickens, phytobiotic additive, production results, meat quality, electronic nose

## Abstract

**Simple Summary:**

Research on broiler feeding has increasingly focused on finding effective alternatives to antibiotic growth promoters (AGPs) due to concerns about antibiotic resistance and antibiotic residues in food products. A total EU-wide ban on AGPs was implemented in 2006. However, 70% of antibiotics are still used in animal treatment, contributing to 700,000 deaths worldwide annually due to antibiotic-resistant infections. As a result, researchers have sought natural alternatives, such as probiotics, prebiotics, enzymes, and plant-based preparations like essential oils, which have shown promise in improving broiler production traits and meat quality. The experiment in this study demonstrated that phytogenic supplements improved the European Production Efficiency Factor (EPEF). Additionally, meat from chickens on the phytogenic diet had better water-holding capacity, higher collagen content, and a favorable profile of amines, ketones, and aldehydes, indicating improved quality. These findings suggest that alternatives to AGPs can enhance broiler chicken production and meat physicochemical properties when used in appropriate doses and proportions.

**Abstract:**

The primary objective of this study was to investigate the impact of a phytogenic additive (PA) in broiler chickens’ diet on production, physiochemical parameters, and the profile of volatile organic compounds present in broiler chickens’ meat. The experiment was conducted in a commercial chicken house, where Ross 308 broiler chickens were divided into two groups, each consisting of 65,000 broilers. One group was fed a diet supplemented with 100 ppm of PA throughout the rearing period. The primary chemical composition of the meat and its physicochemical parameters were determined. A visual assessment of breast muscles for defects and volatile organic compounds were evaluated using an electronic nose system. No statistically significant differences were shown in the production performance of the chickens; while summarizing all production parameters, a higher EPEF index of 31 points in the experimental group was highlighted. Breast muscle quality showed differences in drip loss and WHC (*p* ≤ 0.01) in favor of the experimental group, and a lower cutting force value (*p* ≤ 0.05) was found for breast muscles from the experimental group. The group also had a lower proportion of muscles with a white striping defect, and the results of volatile organic compound profiling showed the most aroma units.

## 1. Introduction

In the last few years, especially in the aspect of broiler feeding, research has focused on finding effective alternatives to antibiotic growth promoters (AGPs) that have both antimicrobial activities and a positive impact on the birds’ production traits and performance, ultimately improving the physicochemical parameters of the meat [1,2]. Antibiotic growth promoters (AGPs) have been used in animal production for almost 50 years [3]. However, with time, the problem regarding antibiotic residues in milk, meat, and eggs and their detrimental effects on human health (allergenic and toxic effects) has received increasing attention. The use of antibiotics as antibiotic growth promoters (AGPs) has been further compounded by the ever-increasing number of pathogens displaying antibiotic resistance, i.e., resistance to antibiotics’ bactericidal or bacteriostatic effects. Given this development, a total EU-wide ban on the use of antibiotics as antibiotic growth promoters (AGPs) in animal production was introduced on 1 January 2006 [4]. Unfortunately, the action taken did not have the desired effect. Nowadays, only 30 percent of the world’s antibiotic production is used to treat humans; the remaining 70 percent is used to treat animals and as substances to stabilize gastrointestinal microbiota composition. There are more than 700,000 deaths worldwide each year that are caused by infections due to pathogenic antibiotic-resistant microorganisms [5], of which approximately 25,000–33,000 are in Europe. The widespread problem of antibiotic resistance in the EU also causes a substantial economic loss of more than EUR 1.5 billion per year related to covering additional healthcare and hospitalization costs [6]. 

The complete ban on antibiotic growth promoters (ASWs) in livestock feeding for slaughter presented animal nutritionists with the daunting challenge of finding alternative feed additives. On the one hand, they had to protect the animal’s gastrointestinal tract from being colonized by pathogenic bacteria and, on the other hand, make the alternative inert to the animal’s body [7]. Based on the results obtained from many experiments, a group of natural and fully effective substitutes for AGPs has been created. The wide range of most commonly used natural replacements for antibiotic growth promoters in the poultry industry includes probiotics and prebiotics (including crude fiber [8,9]), enzymes, preparations to stimulate the immune system, and herb and plant preparations in the form of essential oils, among others [10]. When used as a nutritional supplement in broiler chicken rearing, herbs and plant preparations enhance palatability, stimulate the appetite and regulate the gastrointestinal tract and metabolism. They have a protective function, with anti-diarrheal, anti-bacterial, and anti-inflammatory effects. They also show anabolic effects, reduce susceptibility to stress, strengthen the immune system, and nullify the adverse effects of anti-nutritional substances [11]. Adding phytogenic preparations to the broiler chicken feed mixture improves final production performance [12,13,14] by increasing weight gain, improving the feed conversion ratio (FCR), and improving intestinal morphology [13,14,15,16]. Adding phytogenic preparations, a mixture of equal compositions of thymol and carvacrol at four levels (0, 60, 100, and 200 mg/kg of fed feed) into the broiler chicken feed mixture increases weight gain, improves feed conversion, and decreases feed intake. In addition, the additive used increases the activity of antioxidants and digestive enzymes and improves the immune response, which may positively impact the health and performance of broiler chickens [17,18]. The use of plant-based additives also delays lipid oxidation, directly affecting the quality of the meat [19]. Among the plant preparations used, one of the more popular forms is essential oils, which exhibit antibacterial, antifungal, antiparasitic, and antiviral effects [20]. They belong to a group of chemical compounds with a rather complex chemical structure and diverse impact [21]. The use of essential oils as an additive in the rearing of chicken broilers has positive effects on intestinal function [1,12,22], improved performance [12,19,22], and reduced feed conversion ratio and bird mortality [19].

## 2. Materials and Methods

### 2.1. Animals and Experimental Design

The experiment was conducted in a commercial poultry house. Ross 308 chickens were divided into two groups—control and experimental. The size of each group was 65,000 broiler chickens. Chickens had free access to water and were kept under a controlled light cycle [23]. The birds were kept on straw bedding. The stocking rate in the poultry house at the end of rearing did not exceed 39 kg/m^2^. The experiment was conducted in temperature-controlled rooms, wherein the temperature was gradually reduced from 33 °C ± 0.5 °C during the first week to 22 °C ± 0.3 °C toward the end of the experiment. The chickens were vaccinated on day one against infectious bronchitis (IB) and Newcastle disease (ND) by coarse spray and against infectious bursal disease (IBD) by subcutaneous injection.

During the 39-day rearing period, the chickens were fed a three-phase system based on cereal feed mixtures. The diet was formulated according to the breeding company’s recommendations [23] and comprised a granulated meal of maise and soybean, among other ingredients. The animals were fed with commercial, complete mixes: starter (0–10 days), grower (11–24 days), and finisher (25–39 days). The diets of the chickens are shown in Table 1.

The formulation included five main plant ingredients containing phytoncides to stimulate and support the animals’ natural immunity against bacterial and protozoan infections: turmeric (*Curcuma longa* L.), red pepper (*Capsicum annuum* L.), white mustard (*Sinapis alba* L.), sweet flag (*Acorus calamus* L.), soapwort (*Saponaria officinalis* L.), vegetable oil and palm oil (hydrogenated), aroma (500 mg/100 g), and iron sulphate monohydrate (690 mg Fe/100 g) [24]. The constituents are listed from the composition’s highest to the lowest proportion. The nutrient content of this product was as follows: 88.0% dry matter, 12.6% crude protein, 17.5% crude fiber, 7.0% crude fat, 7.4% crude ash, 0.52% lysine, 0.17% methionine, and 0.03% sodium [24]. In the experimental group, a phytobiotic preparation was added to the feed at a rate of 100 ppm.

Production indicators (FCR, EPEF, body weight, and mortality) were monitored during rearing. A formula was used to calculate the EPEF: (Average grams gained/day × % survival rate)/Feed Conversion × 10.

### 2.2. Sampling Procedures

From each group, 15 males with a body weight close to the average body weight of the group were selected for slaughter. After that, the carcasses were cooled at 4 °C for 24 h, and breast muscles were collected. After the sample had been minced and thoroughly mixed, the collected breast muscles were used for further laboratory tests (determination of chemical and physicochemical composition). The basic chemical composition (water, fat, protein, and collagen) was determined using the NIR method. The determination of the physicochemical composition of the meat consisted of the following parameters: meat pH 24 h after slaughter, own water holding capacity, color parameters (L*, a*, b*), cutting force, free leakage, and thermal loss. An electronic nose utilizing ultrafast gas chromatography for flavor analysis offered a non-destructive, rapid, cost-effective, and reliable technique. This method has been extensively used in scientific research and quality control.

### 2.3. Visual and Tactile Assessment of the Breast Muscle for Quality Defects

Tactile and graphical scale assessments of the occurrence of visual defects in the breast muscle, such as white striping (WS) [25], woody breast (WB) [26], and spaghetti meat (SM) [27], were used to assess for the occurrence of the defect in question.

In WS visual scale:

- 0 = normal—no distinct white lines,
- 1 = moderate—small white lines, generally <1 mm thick but visible on the fillet surface,
- 2 = severe—large white lines (1–2 mm thick) evident on the fillet surface,
- 3 = extremely—thick white bands (>2 mm thickness) covering almost the entire surface of the fillet [25].

In WB tacticle scale:

- 0 = normal breast muscle that is flexible throughout;
- 1 = mild breast muscle that is hard mainly in the cranial region but flexible elsewhere;
- 2 = moderate breast muscle that is hard throughout but flexible in the mid to caudal region;
- 3 = severe muscle that is extremely hard and rigid from the cranial region to the caudal tip [26].

In SM tacticle-visual scale:

- 0 = normal—muscle shows normal consistency and does not display any sign of muscular lesion;
- 1 = moderate—muscle does not show any superficial laceration but has a soft and stringy texture, perceivable by pinching the muscle on its cranial surface;
- 2 = severe—muscle shows extensive superficial lacerations on the cranial and/or caudal surface. Muscle fiber bundles separate from each other, resembling spaghetti pasta [27].

### 2.4. Breast Muscle Color Parameters (L*, a*, b*)

Color parameters were determined twice on whole breast muscle and minced meat using a CR-410 (Konica Minolta, Tokyo, Japan) colorimeter according to the manufacturer’s instructions for the instrument. Each measurement was performed in five replicates, taking their average as the result of the determination. The parameter L* (color lightness) can take values from 0 to 100. a* and b* are trichromaticity coordinates. They can take on positive and negative values. A value of +a* corresponds to red, −a* to green, +b* to yellow, and −b* to blue. The color deviation tolerance ∆E between the color of the breast muscle of broiler chickens in the control and experimental groups was calculated from the formula:

$$∆E=\sqrt{{{\left(L_{1}^{*}-L_{2}^{*}\right)}^{2}+(a_{1}^{*}-a_{2}^{*})}^{2}+{\left(b_{1}^{*}-b_{2}^{*}\right)}^{2}}$$

where:

ΔE—absolute color difference,
$L_{1}^{*}$, $a_{1}^{*}$, $b_{1}^{*}$—color parameters of the breast muscles from chickens from the control group (C),
$L_{2}^{*}$, $a_{2}^{*}$, $b_{2}^{*}$—color parameters of the breast muscles of chickens from the experimental group.

The obtained ΔE values were interpreted according to the scale:

0 < ∆E < 1—standard, invisible color deviation;
< ∆E < 2—minimal deviation, recognizable only by an experienced observer;
2.01 < ∆E < 3.5—mean deviation, recognizable by an inexperienced observer;
3.51 < ∆E < 5—apparent color deviation;
∆E > 5.01—significant color deviation [28].

### 2.5. Determination of Drip Loss and Thermal Loss

To determine drip loss, each left breast muscle was weighed, and then, after 24 h of cold storage, they were dried and weighed again. The difference in weight determined the loss of water. Thermal leakage was determined using the method of Iwiński et al. [29], in which a meat sample of about 5 g was heat-treated at 70 °C for 10 min and cooled for 20 min. Then, the percentage of water lost was determined based on the difference in weight before and after treatment.

### 2.6. Determination of Cutting Force

One sample (1 × 1 × 5 cm) was cut from each group’s ten breast muscles along the muscle fibers. These were used to measure the cutting force using a Zwicki strength testing device, type 1120 (Zwick Roell, Germany), equipped with a Warner-Bratzler cutting element. The value from one measurement was taken as the result of the determination.

### 2.7. Measurement of Breast Muscle pH and WHC, and Determination of Basic Chemical Composition and Collagen Content

After cutting the breast muscles from the carcasses, the samples were prepared for analysis of the chemical composition and physicochemical properties. This involved grinding the right half of the breast muscle once in a laboratory grinder using a mesh with 3 mm diameter holes and mixing the sample thoroughly. In samples prepared this way, the following were determined: pH_24_ using a CP-401 pH-meter with a glass–calomel electrode (Elmetron, Zabrze, Poland). Drip loss and WHC were analyzed according to the method by Michalczuk et al. [30], and the elemental chemical composition of the muscle was analyzed using a near-infrared (NIR) method [30].

### 2.8. Volatile Organic Compounds Profile in Breast Muscle

Analysis of volatile organic compounds (VOCs) present in breast meat was conducted utilizing an electronic nose system, specifically the Heracles II (Alpha M.O.S., Toulouse, France). The methodology employed in this study was adapted from the protocols outlined by Wojtasik-Kalinowska et al. [31] and Górska-Horczyczak et al. [32]. The electronic nose analysis involved ultrafast gas chromatography coupled with headspace sampling. The equipment comprised a detector system featuring two metal columns (which provide two methods of identification and an odor match) with different polarities, namely a nonpolar MXT-5 column and a slightly polar MXT1701 column (10 m × 0.18 mm ID × 0.4 μm film thickness). Additionally, two flame ionization detectors (FIDs) were employed. Kovats indices were determined for two columns based on alkane standards ranging from n-butane to n-hexadecane (Restek, ANCHEM Plus, Warsaw, Poland), measured under identical conditions to the samples. VOC identification was facilitated using AroChemBase (Alpha MOS Co., Toulouse, France), a database encompassing 44,000 compounds with associated sensory descriptors for each compound. Volatile compounds and two retention indices were determined (for the MXT-5 column and the MXT-1701 column). The relative surface area and chemical group were determined for each compound. Each chemical group’s relative peak areas were summed, and sensory descriptors were assigned. Approximately 3 g of breast meat devoid of visible abnormalities or fat tissue was placed into 20 mL headspace vials, which were subsequently capped with Teflon-faced silicone caps. These samples’ vials underwent incubation at 55 °C for 900 s with agitation at 8.33 Hz. Hydrogen was utilized as the carrier gas, circulated at a constant flow rate of 1 mL/min. The injector temperature was set at 200 °C, with an injection volume of 3500 μL and an injection speed of 125 mL/s. The analytes were collected in the trap at 15 °C, then evenly distributed and simultaneously transferred into the two columns. The carrier gas was maintained at a constant pressure of 80 kPa, with a 10 mL/min split flow rate at the column heads. The temperature program of the oven was programmed as follows: initial temperature of 60 °C held for 2 s, followed by a ramp of 3 °C/s to 270 °C, with a subsequent hold for 20 s, and FID1/FID2 operating at 280 °C. The analysis was conducted in six repetitions.

### 2.9. Statistical Analysis

Statistical analyses were performed using SPSS software (PS IMAGO PRO 8.0), and the Student’s T-test was used to determine the significance level between the control and experimental group. If the data did not show a normal distribution, the Mann–Whitney U test was used to determine whether there were significant differences between the study groups. The results were considered statistically significant when associated with a probability lower than *p* ≤ 0.05. Results with a probability lower than *p* ≤ 0.01 were considered highly significant.

## 3. Results and Discussion

The production results of broiler chickens in the control and experimental groups at day 39 are shown in Table 2. The average body weight did not differ between the groups. The European Production Efficiency Factor (EPEF) in the experimental group was 400 and was 31 points higher than that in the control group. The experimental group’s feed conversion ratio was lower by 7.23%. Marć-Pieńkowska et al. [33] observed a significant reduction in FCR and a statistically unconfirmed increase in EPEF when using a vegetable supplement as a mixture of herbs. In their study, Zaikina et al. [34] demonstrated that administering plant feed additives from sweet chestnut wood extract at higher doses to two groups of poultry positively impacted production results. For poultry aged up to 10 days, 650 g per ton of compound feed was administered, while for poultry aged 11 to 34 days or more, 325 g per ton was administered. Additionally, for poultry aged up to 10 days, 800 g per ton of compound feed was given, whereas for poultry aged 11 to 34 days or more, 400 g per ton was administered. The results showed an increased productivity index, decreased feed intake, and a lower feed conversion ratio (FCR). Higher body weights of chickens were achieved, as also indicated by Sakr et al. [35], with the use of mossy paulownia (*Paulownia tomentosa*) leaf extract added to the basal diet in amounts of 0.1, 0.3, and 0.5 mg g/kg of mossy paulownia. Dokou et al. [36] used encapsulated plant extracts (*Origanum vulgare* subsp. *hirtum* L., *Allium sativum* L., *Crithmum maritimum* L., and *Camelina sativa* L.). According to a study by Aljumaah et al. [37], plant additives (e.g., Sangrovit Extra) were mixed into the feed in the form of a mixture of extracts from seven plants. Additionally, a mixture of four acids and a plant extract did not increase performance, and the feed conversion ratio (FCR) was the lowest in chickens aged 1–15 days. Between 15 and 35 days of age, FCR increased in chickens fed with plant additives compared to that when adding an antibiotic or no additive in the feed. In a study by Bello et al. [38], chickens fed with antibiotic growth promoters had significantly higher feed intake. Adding a phytobiotic based on plant extracts (*Zingiber officinale*, *Allium sativum*, *Cichorium intybus*, *Eruca sativa*, *Eucalyptus globulus*, *Trigonella foenum-graecum*) reduced feed intake and lowered the feed conversion ratio (FCR) in chickens aged 22–42 days. In a study by Mehala and Moorthy [39], adding 0.1% turmeric to feed reduced the feed conversion ratio. In contrast, Akbarian et al. [40] indicate that turmeric as an additive has no effect on body weight, feed intake or feed conversion ratio and, consequently, no effect on performance. Black pepper lowered the FCR up to day 7 of chickens’ life. At the end of rearing, black pepper did not affect FCR, gain, feed intake, or performance. In a study by Chodkowska et al. [24], a plant additive with a composition similar to the phytogenic preparation used in the present experiment was used. At 100 mg/kg of feed, it improved the production parameters of broiler chickens.

Table 3 and Table 4 present the breast muscles’ physicochemical parameters and chemical composition. Parameters such as pH_24_ of the meat, thermal loss, and color parameters L* and b* were not significantly different. The experimental group had a lower free leakage rate and self-water-holding capacity; the differences were statistically significant (*p* ≤ 0.01). The cutting force index was lower in the experimental group, and the differences were statistically significant (*p* ≤ 0.05). The color parameter a* was lower in the control group; the difference was statistically significant (*p* ≤ 0.05). The color deviation tolerance ∆E was 1.58 for the experimental group, thus indicating a slight color deviation, recognizable only by an experienced observer. Aljumaah et al. [37] indicate the effect of plant additives on lowering the pH_24_ of meat and increasing the a* parameter. Martinez et al. [41] indicate the impact of the phytobiotic on increasing the a* parameter and decreasing the L* parameter, which coincides with the observations of Zdanowska et al. [42]. In contrast, a linear decrease in the color parameter a* was observed in the study by Kiczorowska et al. [43], while Dokou et al. [36] reported a reduction in the parameter a* and an increase in the parameters L* and b*. Vasilopoulos et al. [44] also reported an increase in the L* and b* parameters and an increase or decrease in the a* parameter depending on the type of extract. In her study, Makała [45] observed no effect of flax seeds on color parameters. The plant additives tested by Aljumaah et al. [37] did not significantly affect parameters such as self-water-holding capacity, thermal loss, or cutting strength. Zdanowska-Sąsiadek et al. [42], on the other hand, found an increase in the pH_24_ of meat, a significant increase or decrease in own-water-holding capacity and a decrease in cutting force, depending on the proportion and composition of the plant preparation used. A reduction in intrinsic water holding capacity due to the addition of the plant preparation to the feed has also been reported by Purwanti et al. [46]. They found no effect on thermal losses, as did Shuvo and Rahman [47]. On the other hand, Sari et al. [48] reported a marked reduction in meat thermal losses when different plant additives were applied to the chickens’ drinking water, and Kiczorowska et al. [43] showed an effect of plant additives to feed on increasing both thermal losses and self-water-holding capacity. Depending on the different treatments of the solutions of various spices used in the experiment of Sari et al. [48], the pH_24_ of the meat was significantly higher or significantly lower than that of chickens watered without additives.

The differences in water, fat, and protein content in the breast muscles of broiler chickens from the experimental group compared to that in the control group were small and not statistically significant. The collagen content of the experimental group was higher than that of the control group, and the difference was statistically significant (*p* ≤ 0.05). Sari et al. [48] demonstrated the effect of plant preparations added to broiler chickens’ drinking water on reducing broiler breast muscles’ water and fat content. Shuvo and Rahman [47] showed that one increased broiler breast muscle’s protein content in a study of two different plant additives. An increase in protein content and a decrease in fat content were observed by Sakr et al. [35] at the highest dose of the plant feed additive. They recorded a reduction in collagen content at the same dose, while its highest content was observed at the lowest dose of the same plant additive. In a study by Martinez et al. [41], phytobiotics increased water and protein content and decreased fat content. In a study by Vasilopoulos et al. [44], the plant additives increased water content but did not affect protein and fat. In their study, the increase in protein content by phytobiotics with amino acid additives was also indicated by Wójcik et al. [49]. Still, there was no effect on water, collagen, or fat content. The lack of effect on breast muscle chemistry was observed by Makała [45] in her study, but she points out the tendency of the applied plant additive to reduce fat content and increase protein content. Similarly, Dokou et al. [36] observed no effect on chemical composition but noted its tendency to reduce fat content.

Table 5 shows the visual assessment of the breast muscles for the presence of white striping (WS—white striations), wooden breast (WB—woody muscles), and spaghetti meat (SM—spaghetti muscles) defects. In the control group, five samples were free of the white striping defect, moderate lesions were observed on seven muscle samples, and severe lesions were observed on three. In the experimental group, eleven samples were defect-free, and moderate lesions were observed in four muscles. The results were not statistically significant. The control or experimental groups did not have wooden breast and spaghetti meat defects. Chodkowska et al. [24] indicate a beneficial effect of the phytogenic preparation on the regeneration and protection of the breast muscle from pathophysiological processes by increasing interleukin-6 content in the blood and decreasing tumor necrosis factor TNF-α concentration in the breast muscle. Dokou et al. [36] observed no effect of the plant supplement on the incidence of wooden breast and white striping defects, similar to Vasilopoulos et al. [44].

The volatile compound composition in the meat product is affected by various factors such as breed, sex, diet, age of the animal, the conditions and process of slaughter, the storage duration, the type of muscle, and meat processing [50]. The results of VOC profiling are presented in Table 6. Seven characteristic chemical compounds were identified: esters, amines, alcohols, aldehydes, ketones, hydrocarbons and terpenes. Statistically, significantly higher values of groups of compounds were observed in the experimental group for amines and ketones (*p* < 0.05). The high protein and free amino acid concentrations in poultry meat provide an ideal setting for forming amines [51]. Ketones can form through lipid autoxidation and microbial metabolism [50]. Concerning aromatic hydrocarbons, many of these compounds often originate from animal feeds [51]. The feed additives in the experimental group resulted in aldehydes, characterized by sensory descriptors such as floral and cinnamon, which may support a better aromatic quality of meat, especially as aroma is thought to be a key factor influencing customer choices when purchasing meat products.

**Figure 1:**
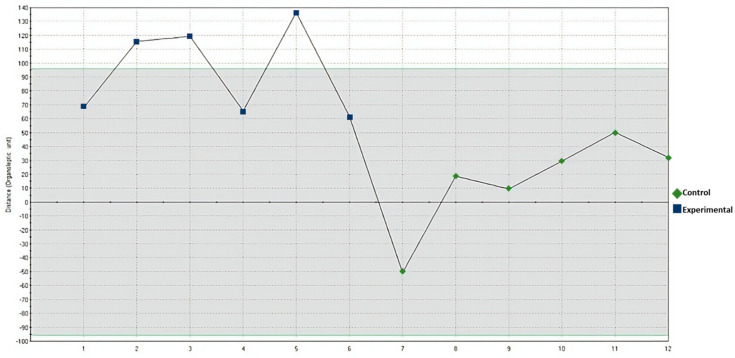
Quality control chart for odor based on straight-line distance.

Figure 1 confirms differences in volatile compound profiles between the control and experimental groups. The analysis shows that the control variant had the fewest aroma units compared to the experimental group (supplemented with PA).

## 4. Conclusions

This experiment investigated the effect of a phytogenic preparation in the diet on the production characteristics and physicochemical properties of broiler chickens’ breast muscles. Using the phytogenic supplement in the chickens’ diet positively affected the European Production Efficiency Factor (EPEF), raising it by 31 points. There was also a positive effect of the chickens’ diet on the physicochemical performance of the breast muscles: the experimental group had a lower free leakage rate and own-water-holding capacity (WHC) (*p* ≤ 0.01). The breast muscles of the chickens in the experimental group had a higher collagen content (*p* ≤ 0.05). Higher values of amines and ketones characterized the experimental group. The feed additive in the experimental group resulted in aldehydes that could be described as floral and cinnamon. In conclusion, it is worth using alternatives to antibiotic growth promoters as they improve broiler chicken meat production characteristics and physicochemical properties in appropriate doses and proportions.

## Figures and Tables

**Table 1:** Compound feeds used in chicken nutrition.

Component g/kg Diet	Starter 1–10 Days	Grower 11–24 Days	Finisher 25–39 Days	Calculated Nutrient Density	Starter 1–10 Days	Grower 11–24 Days	Finisher 25–39 Days
Maise	330.2	326.6	327.2	ME (MJ/kg)	12.39	12.81	13.23
Soybean meal	306.4	300.5	245.6	Crude protein (g/kg)	212.9	209.0	189.3
Wheat	300.0	300.0	350.0	Crude fiber (g/kg)	34.7	34.2	32.1
Oil	28.8	42.7	50.6	Crude fat (g/kg)	51.7	65.4	73.4
Bro1% Robenz	10.0	10.0	10.0	Met. + Cys (g/kg)	8.6	8.4	7.4
Limestone	6.4	4.8	3.5	Threonine (g/kg)	7.7	7.5	6.6
Monocalcium phosphate	7.7	6.0	4.7	Lysine (g/kg)	12.0	11.2	9.8
Choline chloride	2.1	2.4	2.4	Calcium (g/kg)	9.6	8.7	7.9
Methionine	2.8	2.6	2.0	Avail. phosphorus (g/kg)	4.8	4.4	4.0
Lysine	3.5	2.7	2.5				
L-Threonine	1.1	0.9	0.7				
NaHCO_3_	1.0	0.7	0.6				
Optiphose (0.01%) Matrix	0.1	0.1	0.1				

**Table 2:** Production results of broiler chickens.

Parameter	Group	
	Control	Experimental
The average age of slaughter (days)	39	39
Final live weight (kg)	2.55	2.56
EPEF (scores)	369	400
FCR (kg × kg^−1^)	1.66	1.54

EPEF—European Production Efficiency Factor; FCR—feed conversion ratio.

**Table 3:** Physicochemical parameters of the breast muscles.

Indicator	Group				*p*-Value
	Control	SD	Experimental	SD	
**pH_24_**	5.99	0.135	6.10	0.217	0.182
Drip loss (%)	2.01 **	0.513	1.23 **	0.266	<0.001
Thermal leakage (%)	37.87	2.145	37.07	1.082	0.302
WHC (cm^2^/g)	14.90 **	0.619	13.01 **	1.273	0.003
Cutting force (N)	70.33 *	3.081	64.46 *	5.742	0.027
L*	76.17	3.919	77.30	4.860	0.576
a*	8.91 *	0.935	10.01	0.888	0.015
b*	22.49	1.226	22.39	1.365	0.865
∆E	0	-	1.58	-	-

The parameter L* (color brightness) can take values from 0 to 100. The parameters a* and b* are the trichromaticity coordinates. A value of a* corresponds to red color, b* to yellow color; WHC—self-water-holding capacity; SD—standard deviation; ∆E—color deviation tolerance; * superscripted means are significantly different at *p* ≤ 0.05; ** superscripted means are significantly different at *p* ≤ 0.01.

**Table 4:** Chemical composition of breast muscle (%).

Indicator	Group				*p*-Value
	Control	SD	Experimental	SD	
**Water**	74.97	1.305	74.39	0.675	0.287
Fat	3.44	0.156	3.20	0.327	0.056
Protein	21.77	0.670	22.08	0.571	0.293
Collagen	0.83 *	0.044	0.90 *	0.095	0.049

SD—standard deviation; * superscripted means significantly differ at *p* ≤ 0.05.

**Table 5:** Visual assessment of breast muscles for white striping, wooden breast, and spaghetti meat defects.

Indicator	Statistic	Score	Group			
			Control		Experimental	
			%	n	%	n
White striping (WS)		0	33.3	5	73.3	11
		1	46.7	7	26.7	4
		2	20.0	3	0.0	0
Mann–Whitney U test	*p* = 0.060					
Wooden breast (WB)		0	100.0	15	100.0	15
		1	0.0	0	0.0	0
		2	0.0	0	0.0	0
		3	0.0	0	0.0	0
		4	0.0	0	0.0	0
		5	0.0	0	0.0	0
Mann–Whitney U test	-					
Spaghetti meat (SM)		0	100.0	15	100.0	15
		1	0.0	0	0.0	0
		2	0.0	0	0.0	0
Mann–Whitney U test	-					

**Table 6:** Volatile organic compounds in chicken breast meat (relative peak area with the MXT-5 column).

VOCs Group (%) ^$^			Group				*p*-Value
	Control	SD	Sensory Descriptors	Experimental	SD	Sensory Descriptors	
Esters	2.12 **	0.302	agreeable acetone	0.95 **	0.406	agreeable	<0.001
Amines	11.51 **	3.013	alcoholic	45.04 **	13.587	alcoholic	<0.001
Alcohols	45.45 *	5.370	alcoholic	34.96 *	7.262	alcoholic	0.017
Aldehydes	14.60 **	1.934	acetaldehyde cheese earthy	9.02 **	3.626	alkane aldehydic floral cheese earthy cinnamon	0.008
Ketones	4.42 **	0.560	butter	5.47 *	0.814	butter	0.026
Hydrocarbons	0.95 **	0.174	alkane	0.00 **	0.000	-	<0.001
Terpenes	0.79 **	0.143	citrus	0.27 **	0.129	citrus	<0.001

* superscripted means are significantly different at *p* ≤ 0.05; ** superscripted means are significantly different at *p* ≤ 0.01. ^$^ identified based on linear retention index (LRI).

## Data Availability

The original contributions presented in the study are included in the article; further inquiries can be directed to the corresponding author.
